# Is radiotherapy necessary for upper rectal cancer underwent curative resection? A retrospective study of 363 patients

**DOI:** 10.1186/s13014-024-02403-y

**Published:** 2024-01-18

**Authors:** Zhiwei Ma, Jumei Zhou, Ke Liu, Sisi Chen, Qinghui Wu, Lin Peng, Wei Zhao, Suyu Zhu

**Affiliations:** 1 Department of Radiation Oncology, Hunan Cancer Hospital / The Affiliated Cancer Hospital of Xiangya School of Medicine, Central South University, Changsha, 410013 China; 2 Department of Medical Oncology, General Hospital of the Yangtze River Shipping, Wuhan, 430010 China

**Keywords:** Locally advanced rectal cancer, Upper rectal cancer, Risk factors, Radiotherapy, Prognosis analysis

## Abstract

**Background:**

To investigate the impact of radiotherapy (RT) on recurrence and survival in patients with locally advanced upper rectal cancer underwent curative resection.

**Methods:**

363 locally advanced upper rectal cancer cases were identified from the database of our hospital from 2010 to 2018. All patients underwent curative resection and had the lower margin of the tumor located 10–15 cm from the anal verge, among them, 69 patients received pre- or post-operative radiotherapy and 294 patients without. Local control and survivals were compared, and stratification grouping based on European Society for Medical Oncology risk factors were further compared. 1:2 propensity score matching analysis was used to reduce the impact of confounding factors.

**Results:**

There were 207 patients after 1:2 matching (RT group:non-RT group = 69:138). The 5-year overall survival (OS) of the RT group and non-RT group after matching was 84.1% and 80.9%, respectively(*P* = 0.440); the 5-year local recurrence-free survival (LRFS) was 96.5% and 94.7%, respectively(*P* = 0.364); the 5-year distant metastasis-free survival (DMFS) was 76.8% and 76.9%, respectively(*P* = 0.531). Subgroup analysis showed that radiotherapy could not significantly improve the overall survival, local recurrence, and distant metastasis with or without poor prognostic features. In the high-risk subgroup, the 5-year OS was 76.9% and 79.6% for patients treated with radiotherapy and without (*P* = 0.798), LRFS was 94.8% and 94.2%, respectively (*P* = 0.605), DMFS 68.7% and 74.7%, respectively (*P* = 0.233).

**Conclusions:**

Our results suggest that radiotherapy could not improve local control and survival for locally advanced upper rectal cancer patients underwent curative resection, even in the cases with poor prognostic features.

## Background

Colorectal cancer accounts for 10% of all new cancer cases and is the second leading cause (9.4%) of cancer death, and morbidity and mortality continue to rise worldwide [1]. According to the location based on the distance from the anal verge, rectal cancer was categorized as upper, middle, and low. For stage II or III low/middle rectal cancer, preoperative chemoradiotherapy (CRT) is a standard treatment with better LC and sphincter preservation [2]. However, whether upper rectal cancer should be treated with radiotherapy is less certain as it exhibits a better local control and sphincter preservation than those in the low/middle rectum [3, 4]. The available trials and guidelines regarding the management of upper rectal cancers are conflicting. The second analysis of the data from the Swedish and Dutch trials did not show LC benefit from pre-operative RT for upper rectal cancer cases [5, 6], in the contrary, the data from MRC CR07 and NCIC-CTG C016 trial showed that the 3 year local recurrence was as low as 1.2% with preoperative RT and 6.2% for those without for upper rectal cases [7]. The update analysis of upper rectal cancer cases in the CAO/ARO/AIO-94 trial even showed that the improved LC persist over 10 years with preoperative radiation (4.3% vs. 10.4%) [8]. Two large retrospective cohorts analysis from two South Korean cancer centers had drawn opposite conclusion about the benefit of postoperative RT to upper rectal cancer [9, 10]. The current National Comprehensive Cancer Network (NCCN) guideline recommends preoperative concurrent CRT for locally advanced rectal cancer (LARC) without referring to tumor location [11]. The ESMO guideline recommends RT should be implemented to upper rectal cancer patients with poor prognostic features [12]. However, prospective studies focusing particularly on locally advanced upper rectal cancer are lack, and those subsequent analysis from previous studies are old data, there are rare recent studies that evaluate the benefit and the side effects of RT for upper rectal cancer. In addition, with the advancement of radiologic imaging, surgical techniques, radiotherapy, and chemotherapy, the preoperative stage is more accurate, the target volume is more precise, and the side effects of RT are more moderate.

Due to these reasons, the need for the re-evaluation of the significance of radiotherapy in patients with staged II/III upper rectal cancer is mandatory. Therefore, our study focused on patients who visited Hunan Cancer Hospital from 2010 to 2018 diagnosed with stage II/III upper rectal, aimed to evaluate the value of radiotherapy and the relevant factors affecting upper rectal cancer local control and survival.

## Methods

### Study population

We retrospectively reviewed 7109 cases of patients diagnosed with rectal cancer between 2010 and 2018 in Hunan Cancer Hospital. The inclusion criteria included: (1) Assessed by preoperative colonoscopy, the lower margin of the tumor was 10–15 cm from the anal verge; (2) Patients aged 18–80 years; (3) underwent the curative resection and was proved to be adenocarcinoma; (4) diagnosed as clinical stage II or III according to the American Joint Committee on Cancer (AJCC) 7th edition staging system; And exclusion criteria include: (1) previous or concurrent malignancies; (2) progressed, died or lost to follow-up within 3 months after surgery; (3) with distant metastatic diseases at initial presentation; (4) recurrent tumors; (5) relevant clinical data were missing.

### Treatment

Comprehensive staging workups were performed, including digital rectal examination, the chest, abdomen, and pelvis CT (Computed tomography), rectum MRI (Magnetic Resonance Imaging) or rectal ultrasound, carcinoembryonic antigen (CEA) level, complete blood count, liver/renal function tests, colonoscopy with biopsy before treatment decision, pathologically confirmed as adenocarcinoma and radiologically staged as locally advanced upper rectal cancer.

All patients received radical resection of rectal cancer according to the total mesorectal excision (TME) principle. The treatment paradigm included preoperative/postoperative CRT + surgery ± adjuvant chemotherapy (ChT), or surgery ± adjuvant ChT. The decision to treat patients with adjuvant or neoadjuvant RT, the adjuvant ChT and the choice of the ChT regimen was made at the discretion of the surgeon or radiation oncologist. All radiation therapy was performed by 6MV x-ray intensity modulated radiation therapy (IMRT) or volumetric modulated arc therapy (VMRT). Treatment volumes delineation was based on the international consensus reached in 2009 (cases before 2016) and 2016 (cases after 2016) chronically [13, 14]. The dose prescription: pelvis was given a dose of 45 Gy and a sequential tumor (or tumor bed) boost to 50.4 Gy in 1.8 Gy/fx. The concomitant ChT included Capecitabine (825 mg/m^2^) orally twice daily throughout RT or continuous infusion 5-Fluorouracil (400 mg/m^2^/day) plus leucovorin (20 mg/m^2^/day) for 4 days in the first and fifth weeks during the radiation therapy. The adjuvant ChT contained mFOLFOX6 (modified FOLFOX6, oxaliplatin + leucovorin + 5-fluorouracil) or CapeOX (oxaliplatin + capecitabine) implemented before and after RT, six months of adjuvant ChT is preferred.

### Endpoints and follow up

The endpoints were OS, LRFS, DMFS. The OS defined as the time between pathological diagnosis and death or the end of follow-up, LRFS defined as the time between surgery and first locoregional relapse, and DMFS defined as the time between surgery and first distant metastasis. Patients were followed up through outpatient review or telephone contact. Follow-up with physical examination, CT chest/abdomen/pelvis are recommended every 3 months × 2 years, then every 6 months × 3 years and every year after 5 years. Colonoscopy at 1 year and as indicated thereafter. The follow-up deadline was 30 July 2021.

Radiotherapy toxicity was graded from 0 to 4 based on the Radiation Therapy Oncology Group (RTOG) acute radiation injury grade standard [15].

### Statistical analysis

Propensity score matching (PSM) analyses were conducted by R project (version 4.0) for statistical computing and graphics, together with SPSS 22.0 for statistical analyses. Logistic regression was used to estimate the propensity scores, and 1:2 matching without replacement was completed using the nearest neighbor matching principle. All data in our study were categorical data, and the statistical significance of differences was performed by Chi-squared test or Fisher’s exact test. Survival was estimated using the Kaplan–Meier method and log-rank test. Prognostic factors were estimated through univariate and multivariate Cox proportional hazards models. All *P*-values have been two-sided and were considered statistically significant if *P* < 0.05.

## Results

According to the inclusion and exclusion criteria, 363 patients with stage II/III upper rectal cancer were ultimately confirmed, of these, 69 patients received RT, either CRT before (n = 12) or after (n = 57) surgery, and the remaining 294 patients, contained 178 patients had surgery alone and 116 patients had adjuvant ChT after surgery.

### Patients clinical baseline characteristics

The result showed that the RT group was younger (≤ 60 years old, 84.1% vs. 54.4%, *P* < 0.001), have a closer distance from the anal verge (10–12 cm, 91.3% vs. 66.7%, *P* < 0.001), a larger percentage of postoperative pathological stage III (68% vs. 51.7%, *P* = 0.009), and a larger percentage of 4 ~ 6 months adjuvant ChT (53.6% vs. 26.9%, *P* < 0.001). The remaining indicators appeared no significant differences between the two groups (*P* > 0.05). The PSM was applied to minimize the influence of potential confounding factors, there were 69 patients in the RT group and 138 patients in the non-RT group after matching. Overall, the two groups were well matched, except for a higher proportion of 4 ~ 6 months adjuvant chemotherapy (53.6% vs. 26.9%, *P* = 0.017) in the RT group. The baseline characteristics of the two groups before and after matching are listed in Tables 1 and 2.

**Table 1:** Patient characteristic in locally advanced upper rectal cancer before matching

Variable	Overall population	Radiotherapy group (%) (n = 69)	Non-radiotherapy group (%) (n = 294)	*P*-value
*Age*				
≤ 60 years	218	58 (84.1)	160 (54.4)	< 0.001
> 60 years	145	11 (15.9)	134 (45.6)	
*Sex*				
Female	133	27 (39.1)	106 (36.1)	0.678
Male	230	42 (60.9)	188 (63.9)	
*Preoperative CEA(ng/ml)*				
< 5	268	49 (71.0)	219 (74.5)	0.648
≥ 5	95	20 (29.0)	75 (25.5)	
*Distance from anal verge(cm)*				
≤ 12	259	63 (91.3)	196 (66.7)	< 0.001
> 12	104	6 (8.7)	98 (33.3)	
*Pathological differentiation degree*				
High differentiation	26	5 (7.2)	21 (7.1)	0.125
Moderate differentiation	325	59 (85.5)	266 (90.5)	
Low differentiation	12	5 (7.2)	7 (2.4)	
*Circumferential margin status*				
Positive	2	0 (0.0)	2 (0.7)	1.000
Negative	361	69 (100.0)	292 (99.3)	
*Lymphovascular invasion*				
Yes	21	7 (10.1)	14 (4.8)	0.091
No	342	62 (89.9)	280 (95.2)	
*Perineural invasion*				
Yes	16	5 (7.2)	11 (3.7)	0.200
No	347	64 (92.8)	283 (96.3)	
*Number of lymph nodes dissected*				
< 12	222	47 (68.1)	175 (59.5)	0.188
≥ 12	141	22 (31.9)	119 (40.5)	
*pT stage*				
Tis	1	1 (1.4)	0 (0.0)	0.243
T1	5	1 (1.4)	4 (1.4)	
T2	19	6 (8.7)	13 (4.4)	
T3	118	23 (33.3)	95 (32.3)	
T4a	215	37 (53.6)	178 (60.5)	
T4b	5	1 (1.4)	4 (1.4)	
*pN stage*				
N0	158	24 (34.8)	134 (45.6)	0.075
N1	134	24 (34.8)	110 (37.4)	
N2a	46	13 (18.8)	33 (11.2)	
N2b	25	8 (11.6)	17 (5.8)	
*pTNM stage*				
pCR/0	1	1 (1.4)	0 (0.0)	0.009
I	3	2 (2.9)	1 (0.3)	
IIA	57	7 (10.1)	50 (17.0)	
IIB	94	12 (17.4)	82 (27.9)	
IIC	3	0 (0.0)	3 (1.0)	
IIIA	20	5 (7.2)	15 (5.1)	
IIIB	116	21 (30.4)	95 (32.3)	
IIIC	69	21 (30.4)	48 (16.3)	
*4* ~ *6 months adjuvant chemotherapy*				
Yes	116	37 (53.6)	79 (26.9)	< 0.001
No	247	32 (46.4)	215 (73.1)

**Table 2:** Patient characteristic in locally advanced upper rectal cancer after matching

Variable	Overall population	Radiotherapy group (%) (n = 69)	Non-radiotherapy group (%) (n = 138)	*P*-value
*Age*				
≤ 60 years	162	58 (84.1)	104 (75.4)	0.153
> 60 years	45	11 (15.9)	34 (24.6)	
*Sex*				
Female	73	27 (39.1)	46 (33.3)	0.411
Male	134	42 (60.9)	92 (66.7)	
*Preoperative CEA(ng/ml)*				
< 5	148	49 (71.0)	99 (71.7)	0.913
≥ 5	59	20 (29.0)	39 (28.3)	
*Distance from anal verge(cm)*				
≤ 12	194	63 (91.3)	131 (94.9)	0.366
> 12	13	6 (8.7)	7 (5.1)	
*Pathological differentiation degree*				
High/Moderate differentiation	198	64 (92.8)	134 (97.1)	0.164
Low differentiation	9	5 (7.2)	4 (2.9)	
*Circumferential margin status*				
Positive	0	0 (0.0)	0 (0.0)	–
Negative	207	69 (100.0)	138 (100.0)	
*Lymphovascular invasion*				
Yes	16	7 (10.1)	9 (6.5)	0.357
No	191	62 (89.9)	129 (93.5)	
*Perineural invasion*				
Yes	11	5 (7.2)	6 (4.3)	0.512
No	196	64 (92.8)	132 (95.7)	
*Number of lymph nodes dissected*				
< 12	142	47 (68.1)	95 (68.8)	0.916
≥ 12	65	22 (31.9)	43 (31.2)	
*pT stage*				
Tis-T3	93	31 (44.9)	62 (44.9)	1.000
T4	114	38 (55.1)	76 (55.1)	
*pN stage*				
N0-1	153	48 (69.6)	105 (76.1)	0.314
N2	54	21 (30.4)	33 (23.9)	
*pTNM stage*				
pCR-II	81	22 (31.9)	59 (42.8)	0.131
III	126	47 (68.1)	79 (57.2)	
*4* ~ *6 months adjuvant chemotherapy*				
Yes	87	37 (53.6)	50 (36.2)	0.017
No	120	32 (46.4)	88 (63.8)	

### Survival analysis

**Fig. 1:**
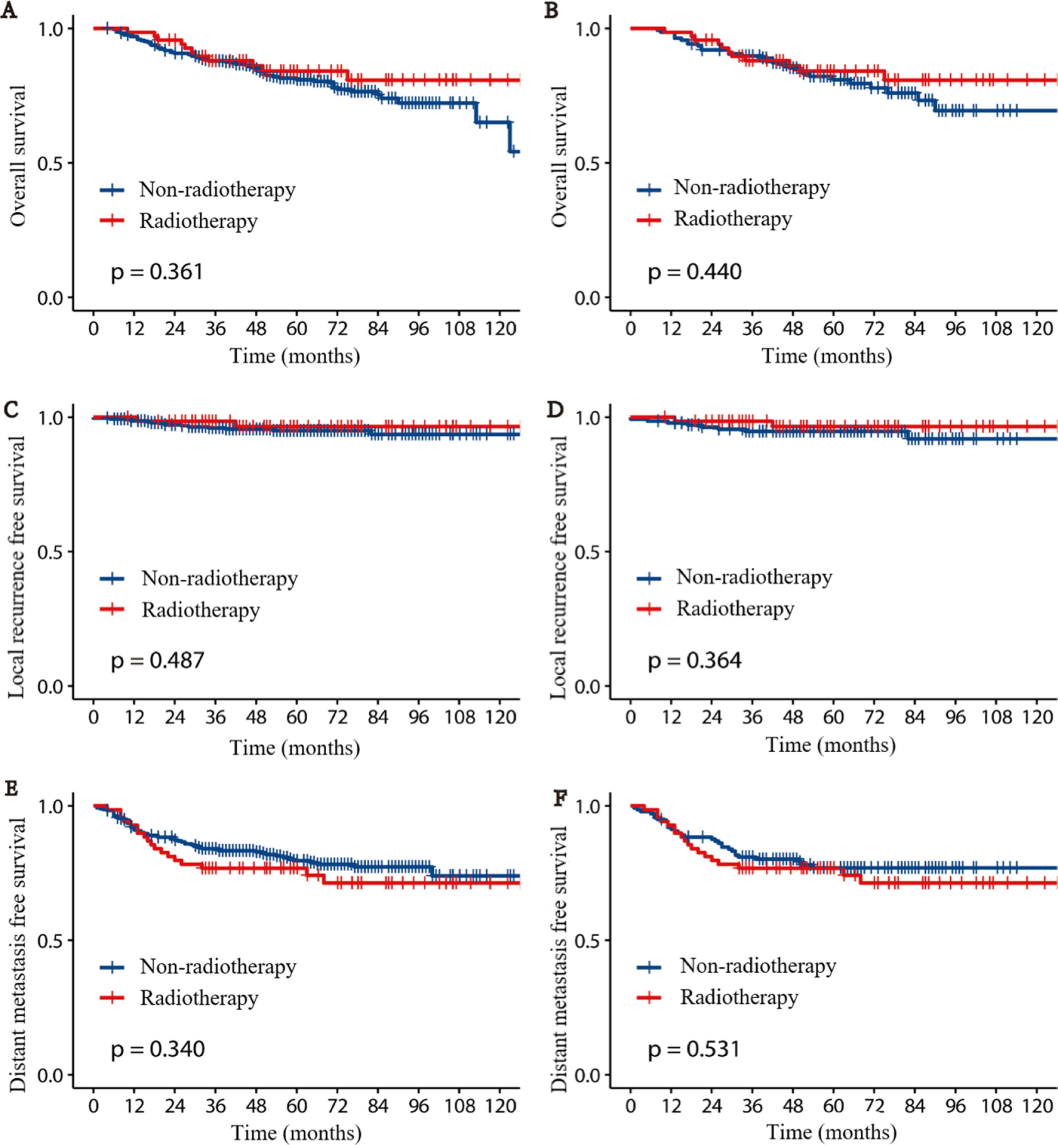
Survival plots for Radiotherapy and Non-radiotherapy patients. cumulative incidence of overall survival (**A**), local recurrence free survival (**C**), distant metastasis free survival (**E**) before matching; cumulative incidence of overall survival (**B**), local recurrence free survival (**D**), distant metastasis free survival (**F**) after matching

Compared with the non-RT group, the RT group exhibited a promising trend in survival benefit and LC, while the difference did not reach statistical significance, besides that, no significant difference was observed in the 5-year DMFS between the two groups. Before matching, the 5-year OS rate was 84.1% and 80.9% in the RT and the non-RT group, respectively (*P* = 0.361), and the corresponding 5-year LRFS was 96.5% and 95.0% (*P* = 0.487), 5-year DMFS was 76.8% and 76.9% (*P* = 0.340). After matching, the 5-year OS rate was 84.1% and 80.9% for patients treated with RT and without, respectively (*P* = 0.440), and the corresponding 5-year LRFS was 96.5% and 94.7% (*P* = 0.364), 5-year DMFS was 76.8% and 76.9% (*P* = 0.531). Figure 1 shows the survival curve for the two groups before and after matching.

### Univariate and multivariate analysis

To further determine the risk factors for survival, recurrence, and metastasis in locally advanced upper rectal cancer, we performed univariate and multivariate Cox regression analysis in 207 patients after PSM. Univariate Cox regression analysis indicated that preoperative CEA levels, pathological differentiation, pathological N stage, and pathological TNM (tumor-node-metastasis) stage were associated with OS (*P* < 0.05), pathological differentiation were associated with LRFS (*P* < 0.05), preoperative CEA levels, pathological N stage, pathological TNM stage were associated with DMFS (*P* < 0.05). We further included variables with *P* ≤ 0.05 in the univariate Cox regression and other potential confounding factors like radiotherapy, the tumor distance from the anal verge, 4 ~ 6 months adjuvant ChT, and pathological T stage into the multivariate Cox regression analysis. Multivariate Cox regression analysis indicated that pathological differentiation (*P* = 0.014), pathological N stage (*P* = 0.006), 4 ~ 6 months adjuvant ChT (*P* = 0.025) were independent prognostic factors for OS, the tumor distance from the anal verge (*P* = 0.029), pathological differentiation (*P* = 0.001) were independent prognostic factors for LRFS, CEA levels (*P* = 0.027), pathological N stage (*P* = 0.004), 4 ~ 6 months adjuvant ChT (*P* = 0.006) were independent prognostic factors for DMFS. More details of univariate and multivariate Cox regression analysis are summarized in Tables 3 and 4.

**Table 3:** Univariate analysis of predictive factors for survival in locally advanced upper rectal cancer patients (n = 207)

Variables	OS HR (95% CI)	*P*-value	LRFS HR (95% CI)	*P*-value	DMFS HR (95% CI)	*P*-value
*Radiotherapy*						
No/yes	0.760 (0.378–1.528)	0.442	0.495 (0.105–2.330)	0.373	1.204 (0.671–2.161)	0.533
*Age*						
≤ 60 / > 60 years	1.413 (0.663–3.011)	0.371	0.034 (0.000–27.822)	0.324	1.623 (0.868–3.035)	0.129
*Sex*						
Female/male	1.078 (0.560–2.074)	0.823	1.354 (0.350–5.240)	0.661	0.920 (0.513–1.651)	0.78
*Pretreatment CEA level*						
< 5ng/ml/ ≥ 5ng/ml	2.182 (1.157–4.115)	0.016	0.133 (0.293–4.384)	0.857	2.230 (1.260–3.948)	0.006
*Distance from the anal verge*						
≤ 12cm/ > 12cm	1.527 (0.469–4.976)	0.482	4.451 (0.937–21.136)	0.06	1.054 (0.327–3.392)	0.93
*Pathological differentiation degree*						
High, moderate/low differentiation	4.166 (1.625–10.680)	0.003	11.655 (2.986–45.495)	< 0.001	2.376 (0.852–6.623)	0.098
*Circumferential margin status*						
Positive/negative	–	–	–	–	–	–
*Lymphovascular invasion*						
Yes/no	0.530 (0.186–1.512)	0.236	0.664 (0.083–5.311)	0.7	0.673 (0.266–1.704)	0.403
*Perineural invasion*						
Yes/no	1.602 (0.218–11.760)	0.643	0.457 (0.057–3.656)	0.46	2.716 (0.374–19.717)	0.323
*Number of lymph nodes dissected*						
< 12/ ≥ 12	1.704 (0.889–3.266)	0.109	1.081 (0.277–4.218)	0.911	1.309 (0.724–2.368)	0.373
*pT stage*						
Tis-T3/T4	1.567 (0.784–3.129)	0.204	1.110 (0.305–4.038)	0.874	1.478 (0.817–2.677)	0.197
*pN stage*						
N0-N1/N2	4.034 (2.145–7.587)	< 0.001	2.158 (0.607–7.671)	0.235	3.445 (1.955–6.073)	< 0.001
*pTNM stage*						
pCR,0-II/III	3.907 (1.636–9.328)	0.002	6.292 (0.797–49.692)	0.081	3.109 (1.506–6.420)	0.002
*4* ~ *6 months adjuvant chemotherapy*						
Yes/no	1.643 (0.832–3.245)	0.153	0.726 (0.210–2.510)	0.613	1.666 (0.905–3.068)	0.101

**Table 4:** Multivariate analysis of predictive factors for survival in locally advanced upper rectal cancer patients (n = 207)

Variables	OS HR (95% CI)	*P*-value	LRFS HR (95% CI)	*P*-value	DMFS HR (95% CI)	*P*-value
*Radiotherapy*						
No/yes	0.780 (0.360–1.691)	0.529	0.226 (0.040–1.292)	0.095	1.409 (0.745–2.664)	0.292
*Pretreatment CEA level*						
< 5ng/ml/ ≥ 5ng/ml	1.880 (0.982–3.598)	0.057	1.377 (0.324–5.855)	0.665	1.926 (1.077–3.442)	0.027
*Distance from the anal verge*						
≤ 12cm/ > 12cm	1.748 (0.506–6.039)	0.377	7.318 (1.219–43.923)	0.029	1.040 (0.310–3.485)	0.95
*Pathological differentiation degree*						
High, moderate/low differentiation	3.636 (1.300–10.170)	0.014	13.154 (2.960–58.455)	0.001	1.453 (0.479–4.403)	0.509
*pT stage*						
Tis-T3/T4	1.346 (0.659–2.750)	0.415	1.320 (0.359–4.849)	0.676	1.132 (0.607–2.112)	0.697
*pN stage*						
N0-N1/N2	2.787 (1.332–5.830)	0.006	0.858 (0.194–3.801)	0.84	2.705 (1.373–5.326)	0.004
*pTNM stage*						
pCR,0-II/III	2.421 (0.894–6.553)	0.082	5.268 (0.569–48.740)	0.143	2.128 (0.917–4.936)	0.079
*4* ~ *6 months adjuvant chemotherapy*						
Yes/no	2.298 (1.109–4.762)	0.025	0.798 (0.216–2.952)	0.735	2.558 (1.315–4.978)	0.006

### Subgroup analysis

**Fig. 2:**
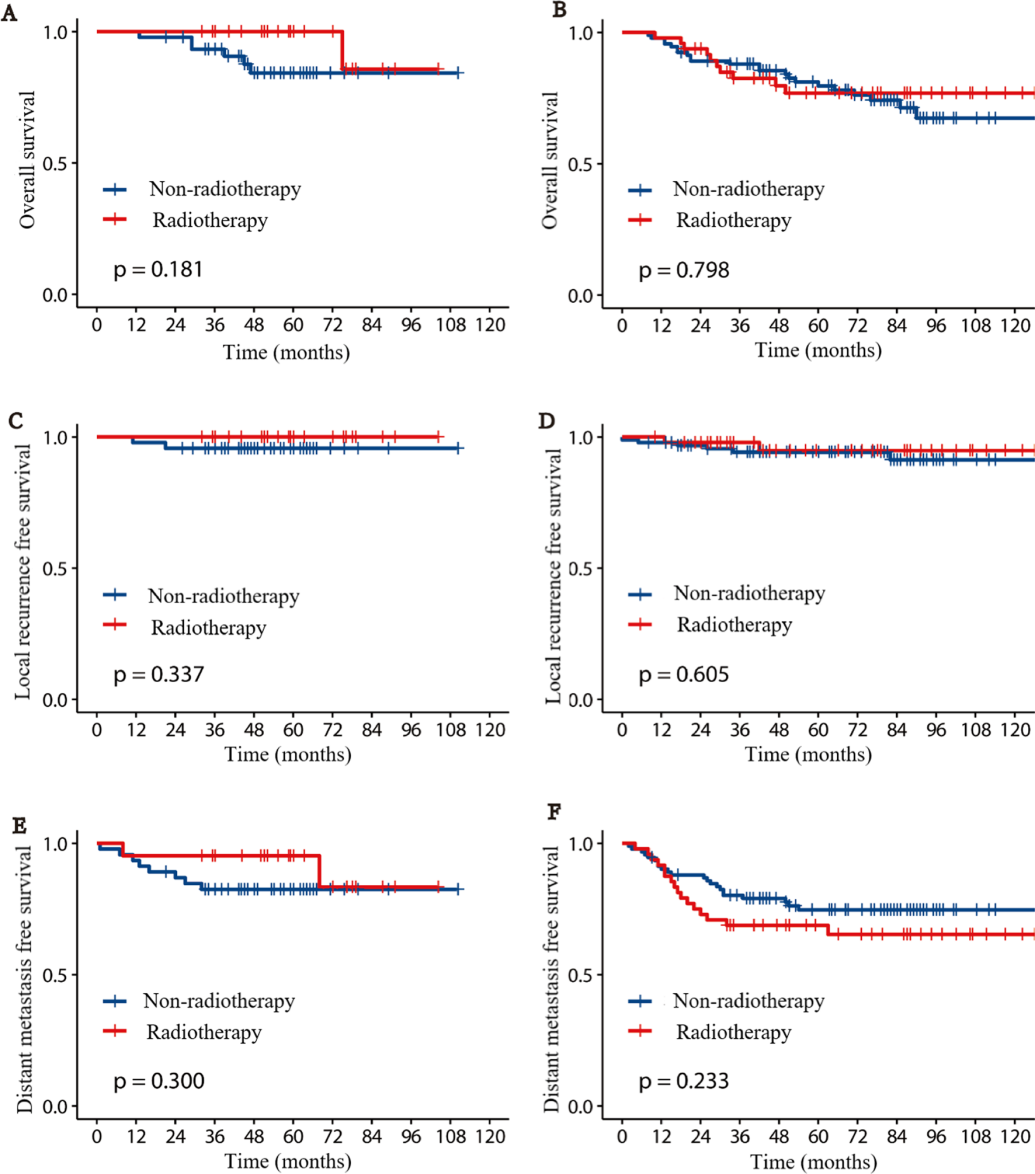
Survival plots based on risk stratification for Radiotherapy and Non-radiotherapy patients after matching. cumulative incidence of overall survival (**A**), local recurrence free survival (**C**), distant metastasis free survival (**E**) in low risk group; cumulative incidence of overall survival (**B**), local recurrence free survival (**D**), distant metastasis free survival (**F**) in high risk group

Subgroup analysis was performed for purpose of identifying the kind of patients that benefit most from RT. We stratified patients according to prognostic factors considering the outcomes of univariate, multivariate Cox regression analysis and ESMO guidelines. 140 patients who have any of the characteristics including poor differentiation, pT4, pN2, involved circumferential resection margin (CRM), vascular invasion, and perineural invasion were classified as high-risk group, including 48 patients treated with RT and 92 patients without, other 67 patients were categorized as low-risk group, including 21 and 46 patients in the RT and the non-RT group, respectively. Subgroup analysis showed that RT could not significantly improve the OS, local and distant control whether in the high-risk group or low-risk group. In the low-risk group, the 5-year OS was 100% and 84.2% in patients who received RT and not, respectively (*P* = 0.181), LRFS was 100% and 95.7%, respectively (*P* = 0.337), DMFS was 95.2% and 82.4%, respectively (*P* = 0.300), and for the high-risk subgroup, the 5-year OS was 76.9% and 79.6% in the RT and non-RT group, respectively (*P* = 0.798), LRFS was 94.8% and 94.2%, respectively (*P* = 0.605), DMFS 68.7% and 74.7%, respectively (*P* = 0.233). Survival analysis curves of the two groups are shown in Fig. 2.

### Adverse effects

For patients with stage II/III upper rectal cancer, RT was well tolerated. Among the 69 patients who received RT, 2 (2.9%) patients developed grade 4 radiation toxicities, both were incomplete intestinal obstruction near the end of RT, and the final RT dose was 46.2Gy/24f and 44Gy/22f, respectively. Grade 3 morbidities occurred in 11 patients (15.9%), including 4 (5.8%) patients with grade 3 hematological toxicity, 6 (8.7%) patients with grade 3 gastrointestinal toxicity, of which 2 patients ended treatment early, the final dose were 24Gy/12f and 30Gy/12f, one patient experienced grade 3 radiation dermatitis. The remaining patients did not have more than grade 2 radiotherapy-related adverse morbidities. All toxicities were controllable, and there were no complication-related deaths.

## Discussion

The location of the rectal tumor has a definite impact on oncological outcomes, the chance of tumor recurrence decreased as the tumor farther from the anus [16, 17], but the current major guidelines for the definition of upper rectal cancer vary considerably, the 10cm-length of the tumor lower tip from the anus and tumor located above the anterior peritoneal reflection were the most frequent landmark used to define as upper rectal cancer, and some studies proposed that anterior peritoneal reflection may be the optimal landmark to identify patients which should avoid radiation [18]. Taking into account that most of the patients do not receive rectal MRI examination when initially diagnosed in our hospital and the referring hospital MRI images were not networked hence could not be reviewed in our archive data base, we defined upper rectal cancers as 10 ~ 15 cm from the anal verge measured by colonoscopy under ESMO guidelines [12].

The wide application of TME surgery and preoperative RT have decreased the 5-year local recurrence to be below 10% in LARC [17], and it's well known that upper rectal cancer has a better prognosis even treated with surgery alone compared to low/middle rectal tumors and sphincter function could be maintained [19, 20]. Thus, it is not entirely clear whether upper rectal cancer patients can benefit further from RT. In the MRC CR07 study, when focused on upper rectal cancer, the 3-year local recurrence rate was 1.2% for patients received RT and 6.2% for patients not (HR = 0.19) [7]. Also, the German rectal cancer group reported lower 5-year local recurrence rate of 2.5% in patients treated with preoperative RT compared to 10.4% in patients treated with surgery alone, the favorable effects of RT persist at 10 years follow-up [2, 8]. However, the Dutch and Swedish trials found that preoperative RT could not significantly decrease local recurrence in upper rectal tumors as observed in low/middle rectal cancer [5, 17].

Our study explores the value of RT based on 363 patients identified as stage II/III upper rectal tumors from 2010 to 2018 in Hunan Cancer Hospital. Considering the two groups were not randomly assigned, PSM was performed to reduce the intergroup confounders. The survival analysis outcomes demonstrated that RT improved the OS and LC, yet the difference did not reach statistical significance, which was consistent with studies at home and abroad. Gao et al. [18] reported 222 cases of above APR cancer patients underwent TME and found no difference in LRFS between 36 cases with RT and 186 cases without. Yoon et al. [10] retrospectively analyzed 263 cases of above 10cm LARC patients underwent TME, and found no difference in OS, DFS (disease-free survival) and LC between adjuvant CRT and ChT alone. Our study showed that the 5-year OS rate was 84.1% and 80.9% in the RT and the non-RT group after matching, respectively (*P* = 0.440), the corresponding 5-year LRFS was 96.5% and 94.7% (*P* = 0.364). Distant metastasis is one of the important reasons for treatment failure, the incidence was reported as high as 25 ~ 40% in LARC, our study observed that RT and ChT are not as effective in preventing distant metastasis, 5-year DMFS was 76.8% and 76.9% after matching, respectively (*P* = 0.531).In addition, the results turn out to be consistent when we have compared surgery plus adjuvant CRT and adjuvant ChT with surgery plus adjuvant ChT, the postoperative RT could not improve LC and survival.

The conflicting results of previous studies, lacking of high-quality prospective trials, and the relatively better prognosis, the side effects related to RT make it difficult to assure whether RT could bring further benefit for locally advanced upper rectal cancer. Thus, identifying appropriate prognostic factors and making radiotherapy choice based on risk stratification are necessary. According to ESMO guidelines, patients with cT4a/b, positive lateral node, extramural vascular invasion, and cT3 with mesorectal fascia (MRF) involved should be treated with RT pre- or post-TME surgery even with upper rectal cancer, besides, RT was recommended when high-quality mesorectal resection cannot be assured for patients with cT3a/b or MRF clear in the high rectum [12]. On the contrary, the NCCN guidelines proposed that observation can be considered when patients underwent radical surgery and proved to be well differentiated, within 2mm from the mesorectum, without lymphatic and venous invasion in pT3N0 upper rectal cancer [11]. A Korean study found that postoperative CRT for upper rectal cancer improved 5-year local recurrence in patients with poor prognostic factors (pT4, pN2, poor differentiation, CRM involvement) compared with surgery alone (96.4% vs. 70.7%, *P* = 0.013) [9]. We reported the outcomes of multivariate Cox regression analysis which demonstrated that pathological differentiation、pathological N stage, 4 ~ 6 months adjuvant ChT were independent prognostic factors for OS, the tumor distance from the anal verge, and the pathological differentiation were independent prognostic factors for LRFS, preoperative CEA levels, pathological N stage, and 4 ~ 6 months adjuvant ChT were independent prognostic factors for DMFS. Patients who have any features of poor differentiation, pT4, pN2, involved CRM, vascular invasion, and perineural invasion were identified as high-risk group, further subgroup analysis showed that RT did not significantly improve the OS, local recurrence, and distant metastasis irrespective of poor prognostic features.

One of the main reasons arousing controversy about RT in upper rectal cancer is the side effects of pelvic RT. In our study, all 69 patients who received RT tolerated well, grade 3 or 4 hematological toxicity, gastrointestinal toxicity, and skin toxicity were 5.8%, 11.6%, and 1.4%, respectively. And there were no complication-related deaths. Treder et al. [21] reported 54 patients with upper rectal cancer to observe the effect of neoadjuvant CRT, stage III patients accounted for 78%, and the grade 3–4 hematology, gastrointestinal, and skin toxicity were 6%, 8%, and 2%, respectively. Thus, the acute toxicity was tolerable considering the high percentage of 68.1% of postoperative radiotherapy in our study which may cause more severe side effects than preoperative radiotherapy, while the late toxicity assessment suffered from missing data.

Constrained by retrospective data, our study has certain limitations. First, only 21.2% of patients had performed MRI examination before operation in our hospital and some patients had done it in referring hospitals, and some had only performed CT examination before operation, which may cause a slight inaccuracy in the preoperative stage. In addition, even though our current study was the biggest one focusing on upper rectal tumors, we cannot further compare the preoperative and postoperative RT groups because of the small sample size (12 patients) of preoperative RT. Third, despite the propensity score matching, there are still uncertain confounders or selection bias caused by its monocentric nature and that long-term outcome data are needed. Therefore, further larger sample sizes and prospective randomized controlled trials are required to clarify the radiotherapy issue.

## Conclusion

In conclusion, for above 10cm locally advanced rectal cancer patients, this study shows that radiotherapy could not improve local control and survival, even in the cases with poor prognostic features.

## Data Availability

All data used and/or analysed during this study are available from the corresponding author on reasonable request.

## Abbreviations

RT: Radiotherapy
LC: Local control
ESMO: European Society for Medical Oncology
OS: Overall survival
LRFS: Local recurrence-free survival
DMFS: Distant metastasis-free survival
CRT: Chemoradiotherapy
NCCN: National Comprehensive Cancer Network
LARC: Locally advanced rectal cancer
AJCC: American Joint Committee on Cancer
CT: Computed tomography
MRI: Magnetic resonance imaging
CEA: Carcinoembryonic antigen
TME: Total mesorectal excision
ChT: Chemotherapy
IMRT: Intensity modulated radiation therapy
VMAT: Volumetric modulated arc therapy
mFOLFOX6: Modified FOLFOX6, oxaliplatin + leucovorin + 5-fluorouracil
CapeOX: Oxaliplatin + capecitabine
RTOG: Radiation Therapy Oncology Group
PSM: Propensity score matching
TNM: Tumor-node-metastasis
CRM: Circumferential resection margin
DFS: Disease-free survival
MRF: Mesorectal fascia
